# (*E*)-1-(4-Bromo­phen­yl)-3-(2-meth­oxy­phen­yl)prop-2-en-1-one

**DOI:** 10.1107/S1600536810026814

**Published:** 2010-07-14

**Authors:** Jerry P. Jasinski, Albert E. Pek, B. Narayana, Prakash K. Kamath, H. S. Yathirajan

**Affiliations:** a Department of Chemistry, Keene State College, 229 Main Street, Keene, NH 03435-2001, USA; b Department of Studies in Chemistry, Mangalore University, Mangalagangotri 574 199, India; c Department of Studies in Physics, Mangalore University, Mangalagangotri 574 199, India; d Department of Studies in Chemistry, University of Mysore, Manasagangotri, Mysore 570 006, India

## Abstract

In the title compound, C_16_H_13_BrO_2_, the dihedral angle between the mean planes of the meth­oxy- and bromo-substituted benzene rings is 24.6 (1)°. The angles between the mean plane of the prop-2-en-1-one group and the 4-bromo­phenyl and 2-meth­oxy­phenyl ring planes are 18.8 (1) and 6.0 (1)°, respectively.

## Related literature

For the use of chalcone compounds or chalcone-rich plant extracts as drugs or food preservatives, see: Dhar (1981 ▶). For the anti-inflammatory, anti­microbial, anti­fungal, anti­oxidant, cytotoxic, and anti­cancer activity of chalcones, see: Dimmock *et al.* (1999 ▶). For their high anti­malarial activity, see: Troeberg *et al.* (2000 ▶). For SHG conversion efficiencies, see: Sarojini *et al.* (2006 ▶). For related structures, see: Arai *et al.* (1994 ▶); Shettigar *et al.* (2006 ▶); Rosli *et al.* (2006 ▶); Ng *et al.* (2006 ▶); Harrison *et al.* (2006 ▶); Patil *et al.* (2007 ▶); Li *et al.* (1992 ▶); Loh *et al.* (2010 ▶). For standard bond lengths, see: Allen *et al.* (1987 ▶).
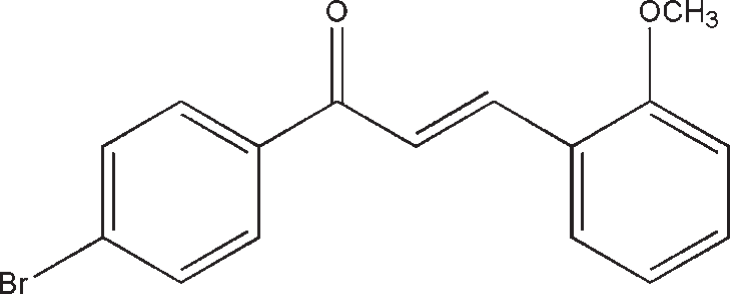


## Experimental

### 

#### Crystal data

C_16_H_13_BrO_2_
*M* *_r_* = 317.17
Monoclinic, 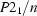

*a* = 17.729 (4) Å
*b* = 4.3505 (9) Å
*c* = 19.335 (4) Å
β = 116.93 (3)°
*V* = 1329.6 (5) Å^3^
*Z* = 4
Mo *K*α radiation
μ = 3.09 mm^−1^
*T* = 100 K
0.55 × 0.50 × 0.35 mm

#### Data collection

Bruker APEXII CCD area-detector diffractometer
Absorption correction: multi-scan (*SADABS*; Bruker, 2008 ▶) *T*_min_ = 0.674, *T*_max_ = 0.746
19884 measured reflections
4121 independent reflections
3635 reflections with *I* > 2σ(*I*)
*R*_int_ = 0.025

#### Refinement

*R*[*F* ^2^ > 2σ(*F* ^2^)] = 0.024
*wR*(*F* ^2^) = 0.070
*S* = 1.32
4121 reflections
173 parameters
H-atom parameters constrained
Δρ_max_ = 0.67 e Å^−3^
Δρ_min_ = −0.22 e Å^−3^

### 

Data collection: *APEX2* (Bruker, 2008 ▶); cell refinement: *SAINT* (Bruker, 2008 ▶); data reduction: *SAINT*; program(s) used to solve structure: *SHELXTL* (Sheldrick, 2008 ▶); program(s) used to refine structure: *SHELXTL*; molecular graphics: *SHELXTL*; software used to prepare material for publication: *SHELXTL*.

## Supplementary Material

Crystal structure: contains datablocks global, I. DOI: 10.1107/S1600536810026814/ci5129sup1.cif

Structure factors: contains datablocks I. DOI: 10.1107/S1600536810026814/ci5129Isup2.hkl

Additional supplementary materials:  crystallographic information; 3D view; checkCIF report

## Figures and Tables

**Fig. 1.**
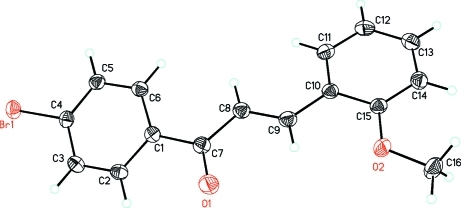
Molecular structure of C16H13BrO2, showing the atom labeling scheme and 50% probability displacement ellipsoids.

## supplementary crystallographic information

### Comment

Chalcones belong to the flavonoid family. Chemically they consist of open-chain flavonoids in which the two aromatic rings are joined by a three-carbon, α-unsaturated carbonyl system. A vast number of naturally occurring chalcones are polyhydroxylated in the aryl rings. The radical quenching properties of the phenolic groups present in many chalcones have raised interest in using the compounds or chalcone rich plant extracts as drugs or food preservatives (Dhar *et al.*, 1981). Chalcones have been reported to possess many useful properties, including anti-inflammatory, antimicrobial, antifungal, antioxidant, cytotoxic, anticancer activities (Dimmock *et al.*, 1999). Many chalcones have been described for their high antimalarial activity (Troeberg *et al.*, 2000). Chalcones are finding applications as organic non-linear optical materials (NLO) due to their good SHG conversion efficiencies (Sarojini *et al.*, 2006). The crystal structures of closely related chalcones, *viz*. 4-bromo-4'-methoxychalcone (Li *et al.*, 1992), 4-bromo-4'-methoxychalcone (Arai *et al.*, 1994), 1-(4-bromophenyl)-3-(4-methoxyphenyl)prop-2-en-1-one (Shettigar *et al.*, 2006), 1-(4-bromophenyl)-3-(2,5-dimethoxyphenyl) prop-2-en-1-one, (Rosli *et al.*, 2006), 1-(4-bromophenyl)-3-(3,4-dimethoxyphenyl)prop-2-en-1-one (Ng *et al.*, 2006), (2E)-3-(1,3-benzodioxol-5-yl)-1-(4-bromophenyl)prop-2-en-1-one (Harrison *et al.*, 2006), and 1-(4-bromophenyl)-3-(3-methoxyphenyl)prop-2-en-1-one (Patil *et al.*, 2007) and (*E*)-1-(6-chloro-2-methyl-4-phenyl-3-quinolyl)-3- (2-methoxyphenyl)prop-2-en-1-one (Loh *et al.*, 2010) have been reported. Hence in continuation with the synthesis and crystal structure determination and also owing to the importance of chalcones, the title new bromo chalcone is synthesized and its crystal structure is reported.

The title compound is a chalcone derivative with 4-bromophenyl and 2-methoxy rings bonded at the opposite ends of a propenone group, the biologically active region (Fig 1). The dihedral angle between the mean planes of the bromo and methoxy substituted benzene rings is 24.6 (1)°. The methoxy (C16—O2—C15—C14 = -2.5 (2)°) substituted benzene ring is virtually planar to the prop-2-en-1-one group (dihedral angle = 6.0 (1)°), whereas the bromo substituted benzene ring attached to the prop-2-en-1-one is slightly twisted (O1—C7—C1—C2 = -16.7 (2)°). Bond distances and angles are in normal ranges (Allen *et al.*, 1987).

### Experimental

To a mixture of 4-bromoacetophenone (0.01 mol, 1.99 g) and 2-methoxybenzaldehyde (0.01 mol, 1.36 g) in 30 ml of ethanol, 7 ml of 30% KOH solution was added. The mixture was stirred for 6 h at room temperature and the precipitate was collected by filtration and purified by recrystallization from ethanol. Single crystals were grown from a acetone-toluene(1:1 v/v) mixture by the slow evaporation method (m.p.329–331 K). Analytical data: found (calculated): C 60.48 (60.59%), H 4.27 (4.13%).

### Refinement

H atoms were placed in their calculated positions and then refined using a riding model with C–H = 0.93–0.96 Å, and with *U*_iso_(H) = 1.18–1.51*U*_eq_(C).

### Crystal data

**Table:**

C_16_H_13_BrO_2_	*F*(000) = 640
*M**_r_* = 317.17	*D*_x_ = 1.584 Mg m^−^^3^
Monoclinic, *P*2_1_/*n*	Mo *K*α radiation, λ = 0.71073 Å
Hall symbol: -p 2yn	Cell parameters from 6810 reflections
*a* = 17.729 (4) Å	θ = 2.4–31.2°
*b* = 4.3505 (9) Å	µ = 3.09 mm^−^^1^
*c* = 19.335 (4) Å	*T* = 100 K
β = 116.93 (3)°	Block, yellow
*V* = 1329.6 (5) Å^3^	0.55 × 0.50 × 0.35 mm
*Z* = 4	

### Data collection

**Table:**

Bruker APEXII CCD area-detector diffractometer	4121 independent reflections
Radiation source: fine-focus sealed tube	3635 reflections with *I* > 2σ(*I*)
graphite	*R*_int_ = 0.025
ω scans	θ_max_ = 31.3°, θ_min_ = 1.3°
Absorption correction: multi-scan (*SADABS*; Bruker, 2008)	*h* = −25→25
*T*_min_ = 0.674, *T*_max_ = 0.746	*k* = −6→6
19884 measured reflections	*l* = −27→28

### Refinement

**Table:**

Refinement on *F*^2^	Primary atom site location: structure-invariant direct methods
Least-squares matrix: full	Secondary atom site location: difference Fourier map
*R*[*F*^2^ > 2σ(*F*^2^)] = 0.024	Hydrogen site location: inferred from neighbouring sites
*wR*(*F*^2^) = 0.070	H-atom parameters constrained
*S* = 1.32	*w* = 1/[σ^2^(*F*_o_^2^) + (0.0363*P*)^2^] where *P* = (*F*_o_^2^ + 2*F*_c_^2^)/3
4121 reflections	(Δ/σ)_max_ = 0.002
173 parameters	Δρ_max_ = 0.67 e Å^−^^3^
0 restraints	Δρ_min_ = −0.22 e Å^−^^3^

### Special details

**Table:**

Geometry. All e.s.d.'s (except the e.s.d. in the dihedral angle between two l.s. planes) are estimated using the full covariance matrix. The cell e.s.d.'s are taken into account individually in the estimation of e.s.d.'s in distances, angles and torsion angles; correlations between e.s.d.'s in cell parameters are only used when they are defined by crystal symmetry. An approximate (isotropic) treatment of cell e.s.d.'s is used for estimating e.s.d.'s involving l.s. planes.
Refinement. Refinement of *F*^2^ against ALL reflections. The weighted *R*-factor *wR* and goodness of fit *S* are based on *F*^2^, conventional *R*-factors *R* are based on *F*, with *F* set to zero for negative *F*^2^. The threshold expression of *F*^2^ > σ(*F*^2^) is used only for calculating *R*-factors(gt) *etc*. and is not relevant to the choice of reflections for refinement. *R*-factors based on *F*^2^ are statistically about twice as large as those based on *F*, and *R*- factors based on ALL data will be even larger.

### Fractional atomic coordinates and isotropic or equivalent isotropic displacement parameters (Å^2^)

**Table:**

	*x*	*y*	*z*	*U*_iso_*/*U*_eq_	
Br1	0.945523 (8)	−0.41655 (3)	0.378517 (7)	0.02597 (6)	
O1	0.68442 (7)	0.3686 (3)	0.49024 (6)	0.0336 (2)	
O2	0.43404 (7)	0.9395 (2)	0.39362 (6)	0.0264 (2)	
C1	0.73529 (8)	0.1076 (3)	0.41311 (7)	0.0189 (2)	
C2	0.81716 (8)	0.0874 (3)	0.47366 (7)	0.0220 (2)	
H2	0.8298	0.1829	0.5207	0.026*	
C3	0.88004 (8)	−0.0719 (3)	0.46513 (7)	0.0231 (3)	
H3	0.9346	−0.0823	0.5056	0.028*	
C4	0.85962 (8)	−0.2161 (3)	0.39466 (7)	0.0205 (2)	
C5	0.77855 (8)	−0.2084 (3)	0.33435 (7)	0.0218 (2)	
H5	0.7657	−0.3108	0.2881	0.026*	
C6	0.71655 (8)	−0.0455 (3)	0.34384 (7)	0.0208 (2)	
H6	0.6618	−0.0383	0.3035	0.025*	
C7	0.67125 (8)	0.2954 (3)	0.42458 (7)	0.0217 (2)	
C8	0.59523 (8)	0.3990 (3)	0.35504 (7)	0.0220 (2)	
H8	0.5869	0.3352	0.3062	0.026*	
C9	0.53875 (9)	0.5816 (3)	0.36129 (7)	0.0228 (2)	
H9	0.5472	0.6243	0.4114	0.027*	
C10	0.46472 (7)	0.7234 (3)	0.29846 (7)	0.0200 (2)	
C11	0.44482 (8)	0.6850 (3)	0.22024 (7)	0.0236 (2)	
H11	0.4792	0.5618	0.2070	0.028*	
C12	0.37500 (9)	0.8268 (3)	0.16228 (7)	0.0261 (3)	
H12	0.3627	0.7990	0.1106	0.031*	
C13	0.32339 (9)	1.0104 (3)	0.18148 (8)	0.0264 (3)	
H13	0.2764	1.1052	0.1424	0.032*	
C14	0.34131 (8)	1.0537 (3)	0.25838 (8)	0.0242 (3)	
H14	0.3065	1.1775	0.2709	0.029*	
C15	0.41145 (8)	0.9114 (3)	0.31666 (7)	0.0204 (2)	
C16	0.38472 (10)	1.1365 (3)	0.41671 (9)	0.0305 (3)	
H16A	0.3883	1.3440	0.4016	0.046*	
H16B	0.4060	1.1269	0.4720	0.046*	
H16C	0.3268	1.0706	0.3920	0.046*	

### Atomic displacement parameters (Å^2^)

**Table:**

	*U*^11^	*U*^22^	*U*^33^	*U*^12^	*U*^13^	*U*^23^
Br1	0.02597 (8)	0.02800 (8)	0.02547 (8)	0.00666 (5)	0.01300 (6)	0.00312 (5)
O1	0.0330 (5)	0.0476 (6)	0.0203 (4)	0.0095 (5)	0.0120 (4)	−0.0023 (4)
O2	0.0279 (5)	0.0312 (5)	0.0211 (4)	0.0059 (4)	0.0120 (4)	−0.0031 (4)
C1	0.0210 (6)	0.0194 (5)	0.0173 (5)	−0.0001 (4)	0.0097 (4)	0.0022 (4)
C2	0.0236 (6)	0.0248 (6)	0.0168 (5)	−0.0009 (5)	0.0086 (5)	0.0000 (4)
C3	0.0215 (6)	0.0266 (6)	0.0189 (5)	0.0007 (5)	0.0071 (5)	0.0027 (4)
C4	0.0236 (6)	0.0185 (5)	0.0217 (5)	0.0022 (4)	0.0122 (4)	0.0037 (4)
C5	0.0262 (6)	0.0204 (6)	0.0187 (5)	0.0006 (5)	0.0101 (5)	−0.0001 (4)
C6	0.0217 (6)	0.0203 (6)	0.0184 (5)	−0.0017 (4)	0.0074 (4)	0.0001 (4)
C7	0.0226 (6)	0.0233 (6)	0.0203 (5)	−0.0004 (5)	0.0108 (4)	0.0001 (4)
C8	0.0222 (6)	0.0245 (6)	0.0193 (5)	−0.0009 (5)	0.0092 (4)	−0.0015 (4)
C9	0.0222 (6)	0.0267 (6)	0.0200 (5)	−0.0026 (5)	0.0100 (5)	−0.0039 (4)
C10	0.0187 (5)	0.0203 (5)	0.0214 (5)	−0.0033 (4)	0.0094 (4)	−0.0028 (4)
C11	0.0253 (6)	0.0236 (6)	0.0234 (6)	−0.0033 (5)	0.0124 (5)	−0.0046 (5)
C12	0.0304 (7)	0.0261 (6)	0.0190 (5)	−0.0047 (5)	0.0089 (5)	−0.0015 (5)
C13	0.0233 (6)	0.0246 (6)	0.0260 (6)	−0.0021 (5)	0.0064 (5)	0.0015 (5)
C14	0.0217 (6)	0.0222 (6)	0.0284 (6)	−0.0010 (5)	0.0110 (5)	−0.0013 (5)
C15	0.0203 (6)	0.0197 (6)	0.0217 (6)	−0.0038 (4)	0.0100 (5)	−0.0031 (4)
C16	0.0345 (7)	0.0325 (7)	0.0310 (7)	0.0052 (6)	0.0206 (6)	−0.0036 (5)

### Geometric parameters (Å, °)

**Table:**

Br1—C4	1.8993 (13)	C8—H8	0.93
O1—C7	1.2243 (16)	C9—C10	1.4611 (18)
O2—C15	1.3600 (15)	C9—H9	0.93
O2—C16	1.4325 (16)	C10—C11	1.3987 (17)
C1—C6	1.3945 (17)	C10—C15	1.4090 (17)
C1—C2	1.3950 (18)	C11—C12	1.383 (2)
C1—C7	1.4946 (17)	C11—H11	0.93
C2—C3	1.3846 (19)	C12—C13	1.386 (2)
C2—H2	0.93	C12—H12	0.93
C3—C4	1.3907 (17)	C13—C14	1.3853 (19)
C3—H3	0.93	C13—H13	0.93
C4—C5	1.3817 (18)	C14—C15	1.3894 (18)
C5—C6	1.3882 (18)	C14—H14	0.93
C5—H5	0.93	C16—H16A	0.96
C6—H6	0.93	C16—H16B	0.96
C7—C8	1.4787 (18)	C16—H16C	0.96
C8—C9	1.3259 (18)		
			
C15—O2—C16	118.42 (11)	C8—C9—H9	116.3
C6—C1—C2	118.74 (12)	C10—C9—H9	116.3
C6—C1—C7	122.47 (11)	C11—C10—C15	118.03 (11)
C2—C1—C7	118.79 (11)	C11—C10—C9	122.71 (12)
C3—C2—C1	121.29 (12)	C15—C10—C9	119.25 (11)
C3—C2—H2	119.4	C12—C11—C10	121.20 (13)
C1—C2—H2	119.4	C12—C11—H11	119.4
C2—C3—C4	118.44 (12)	C10—C11—H11	119.4
C2—C3—H3	120.8	C11—C12—C13	119.82 (12)
C4—C3—H3	120.8	C11—C12—H12	120.1
C5—C4—C3	121.71 (12)	C13—C12—H12	120.1
C5—C4—Br1	118.52 (9)	C12—C13—C14	120.49 (13)
C3—C4—Br1	119.72 (10)	C12—C13—H13	119.8
C4—C5—C6	118.96 (11)	C14—C13—H13	119.8
C4—C5—H5	120.5	C13—C14—C15	119.74 (13)
C6—C5—H5	120.5	C13—C14—H14	120.1
C5—C6—C1	120.82 (12)	C15—C14—H14	120.1
C5—C6—H6	119.6	O2—C15—C14	124.01 (12)
C1—C6—H6	119.6	O2—C15—C10	115.27 (11)
O1—C7—C8	122.06 (12)	C14—C15—C10	120.73 (12)
O1—C7—C1	119.67 (12)	O2—C16—H16A	109.5
C8—C7—C1	118.20 (11)	O2—C16—H16B	109.5
C9—C8—C7	120.98 (12)	H16A—C16—H16B	109.5
C9—C8—H8	119.5	O2—C16—H16C	109.5
C7—C8—H8	119.5	H16A—C16—H16C	109.5
C8—C9—C10	127.46 (12)	H16B—C16—H16C	109.5
			
C6—C1—C2—C3	2.17 (18)	C7—C8—C9—C10	174.50 (12)
C7—C1—C2—C3	−177.12 (11)	C8—C9—C10—C11	−1.3 (2)
C1—C2—C3—C4	−0.75 (18)	C8—C9—C10—C15	179.82 (12)
C2—C3—C4—C5	−1.24 (18)	C15—C10—C11—C12	0.01 (19)
C2—C3—C4—Br1	176.31 (9)	C9—C10—C11—C12	−178.89 (13)
C3—C4—C5—C6	1.73 (18)	C10—C11—C12—C13	−0.1 (2)
Br1—C4—C5—C6	−175.85 (9)	C11—C12—C13—C14	0.2 (2)
C4—C5—C6—C1	−0.24 (18)	C12—C13—C14—C15	−0.2 (2)
C2—C1—C6—C5	−1.67 (18)	C16—O2—C15—C14	−2.45 (19)
C7—C1—C6—C5	177.60 (11)	C16—O2—C15—C10	177.64 (11)
C6—C1—C7—O1	164.07 (13)	C13—C14—C15—O2	−179.85 (12)
C2—C1—C7—O1	−16.66 (18)	C13—C14—C15—C10	0.06 (19)
C6—C1—C7—C8	−18.73 (18)	C11—C10—C15—O2	179.93 (11)
C2—C1—C7—C8	160.54 (11)	C9—C10—C15—O2	−1.13 (17)
O1—C7—C8—C9	1.3 (2)	C11—C10—C15—C14	0.01 (18)
C1—C7—C8—C9	−175.87 (12)	C9—C10—C15—C14	178.95 (11)
